# Supplementary material for: Applying Cell Painting in Non-Tumorigenic Breast Cells to Understand Impacts of Common Chemical Exposures
Source: bioRxiv. 2024 May 3:2024.04.30.591893. Preprint. [Version 1] doi: 10.1101/2024.04.30.591893 (PMC11092634; doi:10.1101/2024.04.30.591893)
Supplement: Supplement 1 [file media-1.pdf]

Small Molecules:

Chemical	CAS Number	Vendor; Catalog number	Solvent
5-Azacytidine	320-67-2	Cayman Chemicals; 11164	Dimethyl Sulfoxide (DMSO)
Decitabine	2353-33-5	Cayman Chemicals; 11166	Dimethyl Sulfoxide (DMSO)
Vorinostat	149647-78-9	Cayman Chemicals; 10009929	Dimethyl Sulfoxide (DMSO)
Valproic Acid	1069-66-5	Cayman Chemicals; 13033	Dimethyl Sulfoxide (DMSO)
Trichostatin A	58880-19-6	Cayman Chemicals; 89730	Dimethyl Sulfoxide (DMSO)
CTPB	586976-24-1	Cayman Chemicals; 19570	Dimethyl Sulfoxide (DMSO)
3- Deazaneplanocin	120964-45-6	Cayman Chemicals; 11102	Dimethyl Sulfoxide (DMSO)
Etoposide	33419-42-0	Cayman Chemicals; 11102	Dimethyl Sulfoxide (DMSO)
Cyclophosphamide	50-18-0	Cayman Chemicals; 13849	Dimethyl Sulfoxide (DMSO)
FCCP	370-86-5	Cayman Chemicals; 15218	Dimethyl Sulfoxide (DMSO)
Diethylstilbestrol	56-53-1	Cayman Chemicals; 10006876	Dimethyl Sulfoxide (DMSO)
Y-27632	146986-50-7	Cayman Chemicals; 10005583	Dimethyl Sulfoxide (DMSO)
CHIR99021	252917-06-9	Cayman Chemicals; 13122	Dimethyl Sulfoxide (DMSO)
Forskolin	66575-29-9	Cayman Chemicals; 11018	Dimethyl Sulfoxide (DMSO)
Prostaglandin E2	363-24-6	Cayman Chemicals;	Dimethyl Sulfoxide

		14010	(DMSO)
Mendione	58-27-5	Cayman Chemicals; M5625	Dimethyl Sulfoxide (DMSO)
Thapsigargin	67526-95-8	Cayman Chemicals; 10522	Dimethyl Sulfoxide (DMSO)
Tunicamycin	11089-65-9	Cayman Chemicals; 11445	Dimethyl Sulfoxide (DMSO)
PMA	16561-29-8	Cayman Chemicals; 10008014	Dimethyl Sulfoxide (DMSO)
FG4592	808118-40-3	Cayman Chemicals; 15294	Dimethyl Sulfoxide (DMSO)
JQ1 (+)	1268524-70-4	Cayman Chemicals; 11187	Dimethyl Sulfoxide (DMSO)

NHANES:

**Chemical**	**CAS Number**	**Vendor; Catalog number**	**Solvent**
Methylparaben	99-76-3	Sigma Aldrich; 47889	Dimethyl Sulfoxide (DMSO)
Propylparaben	94-13-3	Sigma Aldrich; 1577008	Dimethyl Sulfoxide (DMSO)
Thiram	137-26-8	Sigma Aldrich; 45689	Dimethyl Sulfoxide (DMSO)
2,5-Dichlorophenol	583-78-8	Sigma Aldrich; D70007	Dimethyl Sulfoxide (DMSO)
1,4-Dichlorobenzene	99106-46-7	Sigma Aldrich; D56829	Dimethyl Sulfoxide (DMSO)
Sodium (meta) Arsenite	7784-46-5	Sigma Aldrich; S7400	Water
Lead (II) Acetate Trihydrate	6080-56-4	Sigma Aldrich; 316512	Water

Mercury (II) Chloride	7487-94-7	Sigma Aldrich; 215465	Water
Copper (II) Chloride	7447-39-4	Sigma Aldrich; 222011	Water
Cadmium Chloride	10108-64-2	Sigma Aldrich; 202908	Water
Bisphenol S	80-09-1	Sigma Aldrich; 103039	Dimethyl Sulfoxide (DMSO)
Bisphenol A	9980-05-7	Chem Service; S-F7295S	Dimethyl Sulfoxide (DMSO)
PFDA	335-76-2	Sigma Aldrich; 177741	Dimethyl Sulfoxide (DMSO)
PFNA	375-95-1	Sigma Aldrich; 394459	Dimethyl Sulfoxide (DMSO)
DDE	72-55-9	Chem Service; N-10875	Dimethyl Sulfoxide (DMSO)
DDT	8017-34-3	Chem Service; N-11567	Dimethyl Sulfoxide (DMSO)

Supplemental Table S1: Chemical Information: *Small molecules and NHANES chemicals chosen and their associated CAS number, catalog number, vendor and solvents.*

Cellular Stain	Filter (excitation; nm)	Filter (emission; nm)	Cellular component	Cell profiler channel name
Hoechst 33342	350	461	Nucleus	DNA
SYTO 14 Green Fluorescent Nucleic Acid stain	521	547	Nucleoli, Cytoplasmic RNA	RNA
Concanavalin A/Alex Fluor™ 594 conjugate	590	617	Endoplasmic reticulum (α-mannopyranosyl and α-glucopyranosyl residues)	ER
Mito Tracker Deep Red FM	644	665	Mitochondria	Mito
Phalloidin/ Alex Fluor™ Plus 750,<br>Wheat Germ Agglutinin/ CF®770	758/770	784/797	F-Actin cytoskeleton, Golgi apparatus, Plasma membrane	AGP

Supplemental Table S2: Stain Information: *Cell Painting stains selected and their associated fluorophores, and stained cellular compartments.*

Chemical	Compartment	num_upreg	num_downreg	total_num_sig
Azacytidine	Cells	242	238	480
Azacytidine	Cytoplasm	176	232	408
Azacytidine	Nuclei	114	87	201
Bisphenol_A	Cells	11	3	14
Bisphenol_A	Cytoplasm	19	3	22
Bisphenol_A	Nuclei	8	0	8
Bisphenol_S	Cells	39	24	63
Bisphenol_S	Cytoplasm	35	39	74
Bisphenol_S	Nuclei	19	1	20
Cadmium_Chloride	Cells	6	3	9
Cadmium_Chloride	Cytoplasm	5	0	5
Cadmium_Chloride	Nuclei	9	0	9
CHIR99021	Cells	57	22	79
CHIR99021	Cytoplasm	33	10	43
CHIR99021	Nuclei	93	104	197
Copper_Chloride	Cells	163	33	196
Copper_Chloride	Cytoplasm	157	28	185
Copper_Chloride	Nuclei	88	11	99
CTPB	Cells	5	3	8
CTPB	Cytoplasm	6	7	13
CTPB	Nuclei	4	4	8
Cyclophosphamide	Cells	8	12	20
Cyclophosphamide	Cytoplasm	9	7	16
Cyclophosphamide	Nuclei	12	3	15
DCB_14	Cells	0	0	0
DCB_14	Cytoplasm	0	0	0
DCB_14	Nuclei	0	0	0
DCP_25	Cells	0	0	0
DCP_25	Cytoplasm	1	0	1

DCP_25	Nuclei	0	0	0
DDE	Cells	63	36	99
DDE	Cytoplasm	49	1	50
DDE	Nuclei	36	77	113
DDT	Cells	1	5	6
DDT	Cytoplasm	1	3	4
DDT	Nuclei	2	3	5
Deazaneplanocin	Cells	193	256	449
Deazaneplanocin	Cytoplasm	153	240	393
Deazaneplanocin	Nuclei	153	187	340
Decitabine	Cells	155	225	380
Decitabine	Cytoplasm	147	187	334
Decitabine	Nuclei	67	122	189
Diethylstilbestrol	Cells	0	2	2
Diethylstilbestrol	Cytoplasm	13	3	16
Diethylstilbestrol	Nuclei	1	0	1
Estradiol_E2	Cells	11	17	28
Estradiol_E2	Cytoplasm	22	15	37
Estradiol_E2	Nuclei	6	3	9
Etoposide	Cells	79	114	193
Etoposide	Cytoplasm	79	117	196
Etoposide	Nuclei	61	78	139
FCCP	Cells	296	449	745
FCCP	Cytoplasm	284	452	736
FCCP	Nuclei	242	385	627
FG4592	Cells	124	69	193
FG4592	Cytoplasm	124	62	186
FG4592	Nuclei	60	11	71
Forskolin	Cells	13	21	34
Forskolin	Cytoplasm	28	10	38

Forskolin	Nuclei	5	24	29
JQ1	Cells	337	318	655
JQ1	Cytoplasm	307	265	572
JQ1	Nuclei	130	148	278
Lead_Acetate	Cells	49	2	51
Lead_Acetate	Cytoplasm	97	2	99
Lead_Acetate	Nuclei	13	4	17
Menadione	Cells	231	326	557
Menadione	Cytoplasm	250	326	576
Menadione	Nuclei	150	292	442
Mercury_Chloride	Cells	122	5	127
Mercury_Chloride	Cytoplasm	113	5	118
Mercury_Chloride	Nuclei	99	1	100
Methylparaben	Cells	0	0	0
Methylparaben	Cytoplasm	0	0	0
Methylparaben	Nuclei	0	0	0
PFDA	Cells	0	0	0
PFDA	Cytoplasm	0	0	0
PFDA	Nuclei	0	0	0
PFNA	Cells	0	2	2
PFNA	Cytoplasm	0	2	2
PFNA	Nuclei	0	0	0
PMA	Cells	488	398	886
PMA	Cytoplasm	466	336	802
PMA	Nuclei	369	176	545
Propylparaben	Cells	0	0	0
Propylparaben	Cytoplasm	2	0	2
Propylparaben	Nuclei	0	0	0
Prostaglandin_E2	Cells	4	27	31
Prostaglandin_E2	Cytoplasm	3	42	45

Prostaglandin_E2	Nuclei	3	2	5
Sodium_Arsenite	Cells	112	4	116
Sodium_Arsenite	Cytoplasm	99	2	101
Sodium_Arsenite	Nuclei	113	2	115
Thiram	Cells	287	143	430
Thiram	Cytoplasm	239	131	370
Thiram	Nuclei	269	94	363
Trichostatin_A	Cells	235	291	526
Trichostatin_A	Cytoplasm	229	272	501
Trichostatin_A	Nuclei	150	195	345
Tunicamycin	Cells	254	335	589
Tunicamycin	Cytoplasm	264	335	599
Tunicamycin	Nuclei	235	305	540
Valproic_acid	Cells	0	0	0
Valproic_acid	Cytoplasm	0	0	0
Valproic_acid	Nuclei	1	2	3
Vorinostat	Cells	347	287	634
Vorinostat	Cytoplasm	276	269	545
Vorinostat	Nuclei	128	160	288
Y27632	Cells	89	34	123
Y27632	Cytoplasm	98	43	141
Y27632	Nuclei	52	40	92

Supplemental Table S3: Chemical Comparisons in Regions of Interest (ROIs): *Significant features up /downregulated in Nucleus, Cells and Cytoplasm.*

Azacytidine	AGP	18	37	55
Azacytidine	DNA	106	221	327
Azacytidine	ER	62	55	117
Azacytidine	Mito	163	12	175
Azacytidine	RNA	122	164	286
Bisphenol_A	AGP	5	0	5
Bisphenol_A	DNA	0	0	0
Bisphenol_A	ER	20	0	20
Bisphenol_A	Mito	9	0	9
Bisphenol_A	RNA	4	0	4
Bisphenol_S	AGP	2	0	2
Bisphenol_S	DNA	37	54	91
Bisphenol_S	ER	15	0	15
Bisphenol_S	Mito	10	0	10
Bisphenol_S	RNA	18	5	23
Cadmium_Chloride	AGP	2	0	2
Cadmium_Chloride	DNA	0	0	0
Cadmium_Chloride	ER	11	0	11
Cadmium_Chloride	Mito	0	0	0
Cadmium_Chloride	RNA	6	2	8
CHIR99021	AGP	5	0	5
CHIR99021	DNA	6	0	6
CHIR99021	ER	1	7	8
CHIR99021	Mito	101	22	123
CHIR99021	RNA	49	91	140
Copper_Chloride	AGP	20	0	20
Copper_Chloride	DNA	7	0	7
Copper_Chloride	ER	97	8	105
Copper_Chloride	Mito	217	26	243
Copper_Chloride	RNA	45	7	52
CTPB	AGP	0	0	0
CTPB	DNA	1	0	1

CTPB	ER	0	7	7
CTPB	Mito	0	0	0
CTPB	RNA	4	0	4
Cyclophosphamide	AGP	3	0	3
Cyclophosphamide	DNA	1	0	1
Cyclophosphamide	ER	3	3	6
Cyclophosphamide	Mito	0	8	8
Cyclophosphamide	RNA	12	4	16
DCB_14	AGP	0	0	0
DCB_14	DNA	0	0	0
DCB_14	ER	0	0	0
DCB_14	Mito	0	0	0
DCB_14	RNA	0	0	0
DCP_25	AGP	1	0	1
DCP_25	DNA	0	0	0
DCP_25	ER	0	0	0
DCP_25	Mito	0	0	0
DCP_25	RNA	0	0	0
DDE	AGP	0	0	0
DDE	DNA	0	0	0
DDE	ER	70	0	70
DDE	Mito	0	0	0
DDE	RNA	75	107	182
DDT	AGP	3	7	10
DDT	DNA	0	0	0
DDT	ER	0	0	0
DDT	Mito	0	0	0
DDT	RNA	0	0	0
Deazaneplanocin	AGP	15	16	31
Deazaneplanocin	DNA	121	301	422
Deazaneplanocin	ER	20	52	72

Deazaneplanocin	Mito	137	16	153
Deazaneplanocin	RNA	136	232	368
Decitabine	AGP	13	0	13
Decitabine	DNA	83	137	220
Decitabine	ER	22	43	65
Decitabine	Mito	77	9	86
Decitabine	RNA	125	285	410
Diethylstilbestrol	AGP	14	4	18
Diethylstilbestrol	DNA	0	1	1
Diethylstilbestrol	ER	0	0	0
Diethylstilbestrol	Mito	0	0	0
Diethylstilbestrol	RNA	0	0	0
Estradiol_E2	AGP	0	0	0
Estradiol_E2	DNA	8	4	12
Estradiol_E2	ER	15	25	40
Estradiol_E2	Mito	0	0	0
Estradiol_E2	RNA	12	6	18
Etoposide	AGP	12	27	39
Etoposide	DNA	112	254	366
Etoposide	ER	10	0	10
Etoposide	Mito	52	1	53
Etoposide	RNA	1	4	5
FCCP	AGP	164	340	504
FCCP	DNA	113	261	374
FCCP	ER	230	63	293
FCCP	Mito	133	221	354
FCCP	RNA	132	341	473
FG4592	AGP	0	0	0
FG4592	DNA	31	12	43
FG4592	ER	9	0	9
FG4592	Mito	222	54	276

FG4592	RNA	40	45	85
Forskolin	AGP	6	0	6
Forskolin	DNA	23	4	27
Forskolin	ER	9	41	50
Forskolin	Mito	0	0	0
Forskolin	RNA	0	0	0
JQ1	AGP	14	6	20
JQ1	DNA	111	240	351
JQ1	ER	203	58	261
JQ1	Mito	248	58	306
JQ1	RNA	126	292	418
Lead_Acetate	AGP	21	6	27
Lead_Acetate	DNA	0	0	0
Lead_Acetate	ER	63	0	63
Lead_Acetate	Mito	64	0	64
Lead_Acetate	RNA	11	0	11
Menadione	AGP	116	287	403
Menadione	DNA	100	209	309
Menadione	ER	89	157	246
Menadione	Mito	140	9	149
Menadione	RNA	124	213	337
Mercury_Chloride	AGP	19	1	20
Mercury_Chloride	DNA	0	0	0
Mercury_Chloride	ER	139	1	140
Mercury_Chloride	Mito	149	2	151
Mercury_Chloride	RNA	26	0	26
Methylparaben	AGP	0	0	0
Methylparaben	DNA	0	0	0
Methylparaben	ER	0	0	0
Methylparaben	Mito	0	0	0
Methylparaben	RNA	0	0	0

PFDA	AGP	0	0	0
PFDA	DNA	0	0	0
PFDA	ER	0	0	0
PFDA	Mito	0	0	0
PFDA	RNA	0	0	0
PFNA	AGP	0	2	2
PFNA	DNA	0	0	0
PFNA	ER	0	0	0
PFNA	Mito	0	0	0
PFNA	RNA	0	0	0
PMA	AGP	325	166	491
PMA	DNA	87	193	280
PMA	ER	375	148	523
PMA	Mito	363	170	533
PMA	RNA	105	148	253
Propylparaben	AGP	0	0	0
Propylparaben	DNA	2	0	2
Propylparaben	ER	0	0	0
Propylparaben	Mito	0	0	0
Propylparaben	RNA	0	0	0
Prostaglandin_E2	AGP	2	0	2
Prostaglandin_E2	DNA	0	7	7
Prostaglandin_E2	ER	0	0	0
Prostaglandin_E2	Mito	5	40	45
Prostaglandin_E2	RNA	2	22	24
Sodium_Arsenite	AGP	17	0	17
Sodium_Arsenite	DNA	0	0	0
Sodium_Arsenite	ER	116	1	117
Sodium_Arsenite	Mito	170	1	171
Sodium_Arsenite	RNA	18	0	18
Thiram	AGP	130	37	167
Thiram	DNA	89	113	202

Thiram	ER	312	100	412
Thiram	Mito	205	41	246
Thiram	RNA	24	24	48
Trichostatin_A	AGP	79	128	207
Trichostatin_A	DNA	121	245	366
Trichostatin_A	ER	178	31	209
Trichostatin_A	Mito	48	24	72
Trichostatin_A	RNA	121	263	384
Tunicamycin	AGP	76	95	171
Tunicamycin	DNA	53	114	167
Tunicamycin	ER	112	228	340
Tunicamycin	Mito	326	119	445
Tunicamycin	RNA	135	347	482
Valproic_acid	AGP	0	2	2
Valproic_acid	DNA	0	0	0
Valproic_acid	ER	0	0	0
Valproic_acid	Mito	1	0	1
Valproic_acid	RNA	0	0	0
Vorinostat	AGP	84	80	164
Vorinostat	DNA	84	185	269
Vorinostat	ER	132	57	189
Vorinostat	Mito	260	28	288
Vorinostat	RNA	122	282	404
Y27632	AGP	208	93	301
Y27632	DNA	0	0	0
Y27632	ER	24	12	36
Y27632	Mito	0	7	7
Y27632	RNA	0	0	0

Supplemental Table S4: Chemical Comparisons by individual stains: *Significant features up/downregulated in DNA, RNA, ER, Mito and AGP*.

Chemical Comparison		N = 3042 total features	Feature Compartment (N = 3042)			Feature Stain (N = 2,811*)				
**Chemical 1 (N = # significant figures)**	**Chemical 2 (N = # significant figures)**	**# of shared significant features (one-sided ficher's test p-value)**	**Cells N = 1059 total features**	**Cytoplasm N = 1026 total features**	**Nuclei N = 957 total features**	**DNA N = 521 total features**	**RNA N = 575 total features**	**Mito N = 578 total features**	**ER N = 568 total features**	**AGP N = 569 total features**
DDE (N = 262)	CHIR99021 (N = 293)	122 (5.55E-63)	19 (2.83 E-06)	0	103 (9.17E-81)	0	122 (7.52E-76)	0	0	0
Thiram (N = 1163)	PMA (N = 2233)	1070 (6.78E-246)	415 (2.06 E-95)	356 (1.41E-85)	299 (2.86E-81)	181 (1.87 E-73)	45 (3.31E-17)	215 (1.18E-21)	399 (4.80E-143)	157 (5.55E-22)
Copper_Chloride (N = 480)	FG4592 (N = 450)	243 (7.31E-114)	99 (7.73 E-40)	97 (2.21E-41)	47 (3.82E-37)	0	10 (0.06)	219 (1.54E-120)	4 (0.013)	0
Sodium_Arsenite (N = 332)	Mercury_Chloride (N = 345)	293 (3.28E-321)	108 (7.97 E-125)	97 (2.08E-116)	88 (6.75E-93)	0	18 (<E-300)	143 (7.49E-116)	113 (8.70E-100)	15 (1.09E-26)
Copper_Chloride (N = 480)	Mercury_Chloride (N = 345)	275 (6.70E-207)	104 (5.87 E-88)	106 (2.71E-88)	65 (4.30E-54)	0	21 (4.01E-22)	142 (1.01E-73)	91 (1.61E-64)	18 (1.52E-33)
Copper_Chloride (N = 480)	Sodium_Arsenite (N = 332)	258 (4.39E-186)	103 (4.62 E-82)	96 (3.19E-85)	59 (2.89E-39)	0	17 (4.21E-21)	141 (1.60E-56)	80 (2.04E-54)	17 (<E-300)

Supplemental Table S5: Overlap of dose dependent features: *Statistical significance of the overlap of dose-dependent features shared between two chemicals, typically a validated small molecule and a NHANES prioritized chemical.*

**Chemical Name**	**NHANES Biomarker**	**Chemical Codename**
Lead (II) Acetate Trihydrate	Blood lead (ug/dL)	LBXBPB
Copper (II) Chloride	Serum Copper (ug/dL)	LBXSCU
Sodium (meta) Arsenite	Urinary arsenic, total (ug/L)	URXUAS
Mercury (II) Chloride	Blood mercury, total (ug/L)	LBXTHG
Cadmium Chloride	Blood cadmium (ug/L)	LBXBCD
Bisphenol A	Urinary Bisphenol A (ng/mL)	URXBPH
Bisphenol S	Urinary Bisphenol S (ug/L)	URXBPS
Methylparaben	Methyl paraben (ng/ml)	URXMPB
Propylparaben	Propyl paraben (µg/L)	URXPPB
PFDA	Perfluorodecanoic acid	LBXPFDE
PFNA	Perfluorononanoic acid	LBXPFNA
Thiram	Urinary 2-Thioxothiazolidine-4-carboxylic acid (ng/mL)	URXTTC
2,5-Dichlorophenol	Urinary 2,5-dichlorophenol (µg/L)	URX14D
1,4-Dichlorobenzene	Blood 1,4-Dichlorobenzene (ng/mL)	LBXVDB
DDE	ppDDE Lipid Adjusted (ng/g)	LBXPDELA
DDT	ppDDT lipid Adj (ng/g)	LBXPDELA

Supplemental Table S6: NHANES Biomarker Information: *Chemical used in-vitro and their corresponding exposure biomarker codenames in NHANES.*

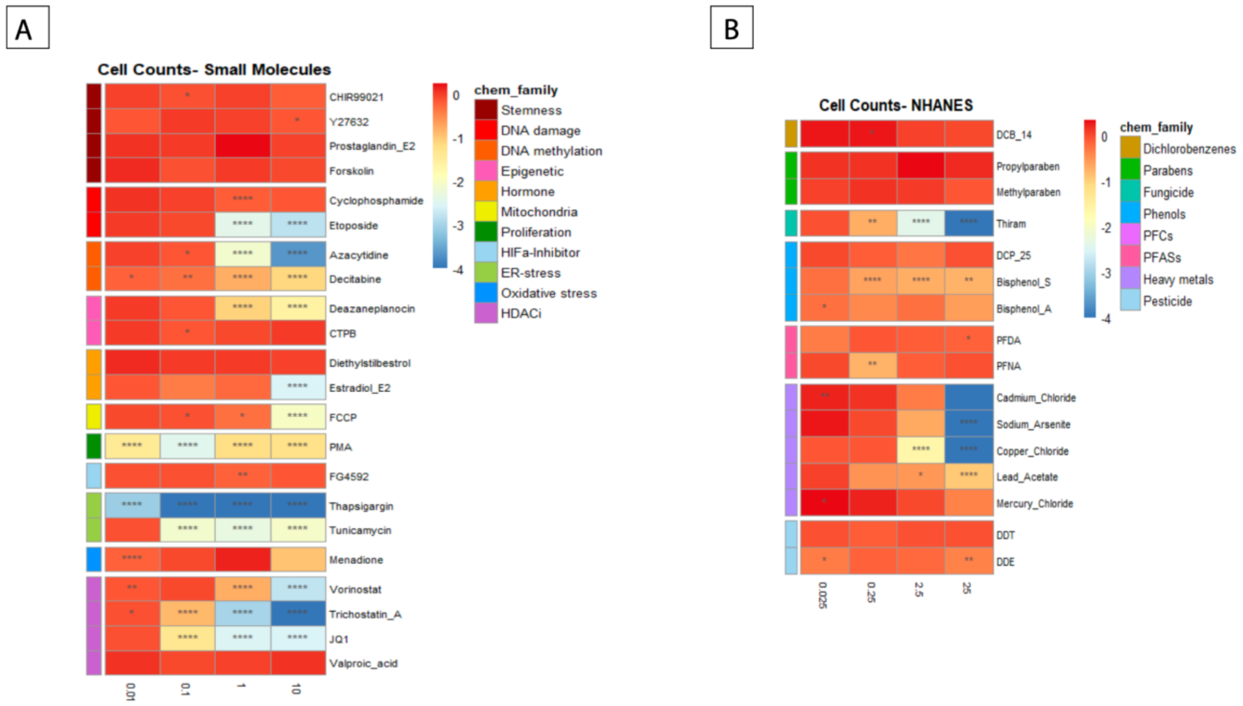

Supplementary Figure S1: Cell Counts: *A heatmap representation of the cell counts normalized by control cell counts for the tested small molecules and NHANES chemicals. P values were determined using the Wilcoxon test. *P<0.05, **P<0.01, ****P<0.0001*

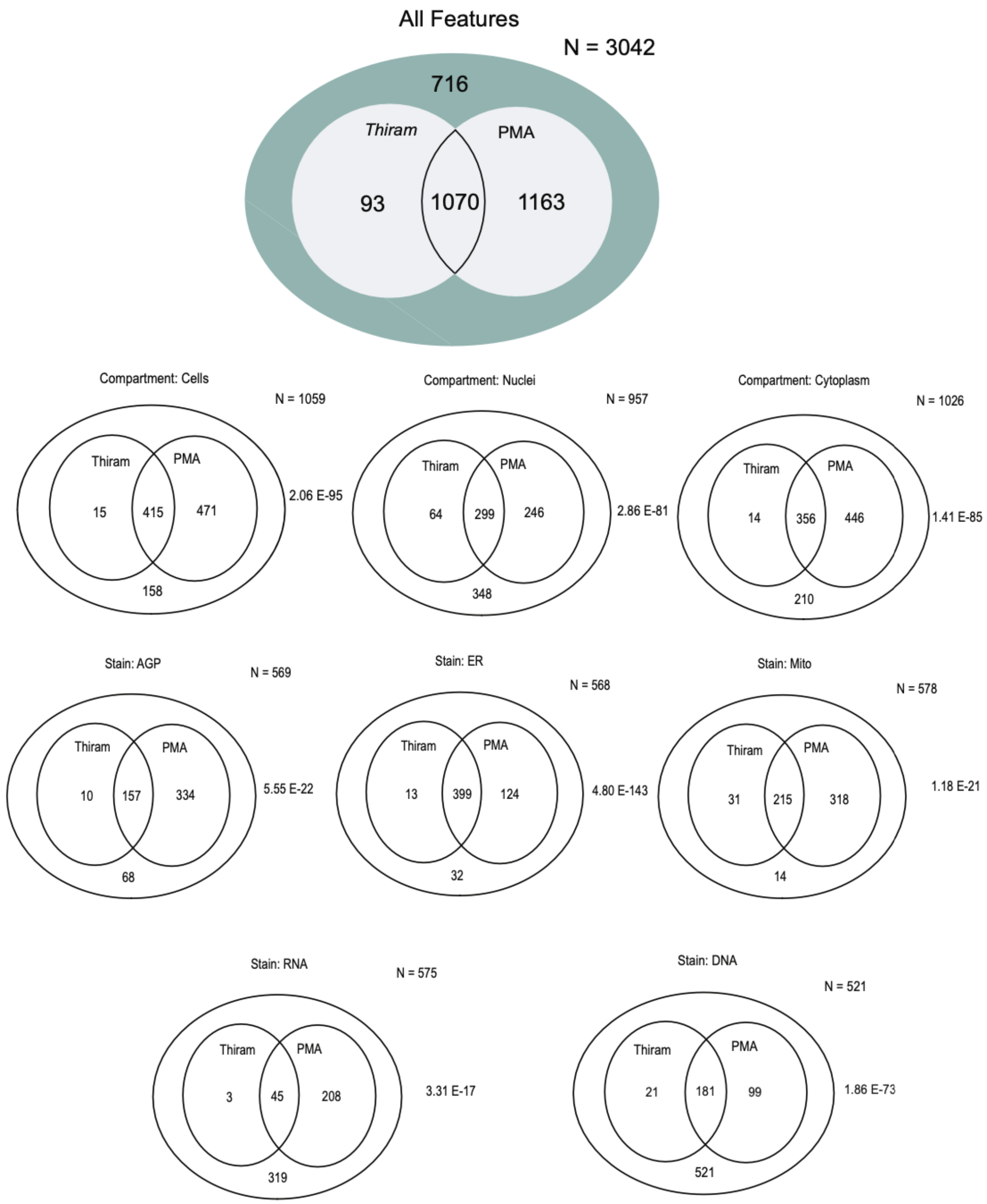

Supplementary Figure S2: Overlap between Thiram and PMA: *A Venn diagram representing the overlap of common features between 2 chemical combinations: PMA and Thiram within different Regions of Interest: Cells, Nuclei and Cytoplasm along with the significant cellular stain showing maximum overlapping features.*
